# Supplementary material for: Caspase-1 initiates apoptosis in the absence of gasdermin D
Source: Nat Commun. 2019 May 7;10:2091. doi: 10.1038/s41467-019-09753-2 (PMC6505044; doi:10.1038/s41467-019-09753-2)
Supplement: Supplementary file 1 — Supplementary Information [file 41467_2019_9753_MOESM1_ESM.pdf]

Supplementary Information for

Caspase-1 initiates apoptosis in the absence of gasdermin D

Tsuchiya et al.

Supplementary Table 1. Oligonucleotides used in this study

Oligonucleotide	Sequence	Method
mGsdmd_gRNA_1_top	CACCGCAGCATCCTGGCATTCCGAG	CRISPR/Cas9-mediated genome editing
mGsdmd_gRNA_1_bottom	AAACCTCGGAATGCCAGGATGCTGC	CRISPR/Cas9-mediated genome editing
mGsdmd_gRNA_2_top	CACCGCAACAGCTTCGGAGTCGTG	CRISPR/Cas9-mediated genome editing
mGsdmd_gRNA_2_bottom	AAACCACGACTCCGAAGCTGTTGC	CRISPR/Cas9-mediated genome editing
mGsdme_gRNA_1_top	CACCGTGGAGAGTCACTCTTCGTT	CRISPR/Cas9-mediated genome editing
mGsdme_gRNA_1_bottom	AAACAACGAAGAGTGACTCTCCAC	CRISPR/Cas9-mediated genome editing
mGsdme_gRNA_2_top	CACCGTCCCAATAGCCCCGCTCTTA	CRISPR/Cas9-mediated genome editing
mGsdme_gRNA_2_bottom	AAACTAAGAGCGGGGCTATTGGGAC	CRISPR/Cas9-mediated genome editing
mBid_gRNA_1_top	CACCGGTCAGCAACGGTTCCGGCC	CRISPR/Cas9-mediated genome editing
mBid_gRNA_1_bottom	AAACGGCCGGAACCGTTGCTGACC	CRISPR/Cas9-mediated genome editing
mBid_gRNA_2_top	CACCGCCAGCCGCTCCTTCAACCA	CRISPR/Cas9-mediated genome editing
mBid_gRNA_2_bottom	AAACTGGTTGAAGGAGCGGCTGGC	CRISPR/Cas9-mediated genome editing
mCasp2_gRNA_1_top	CACCGAGTCTGTGACCCGGTGTGC	CRISPR/Cas9-mediated genome editing
mCasp2_gRNA_1_bottom	AAACGCACACCGGGTCACAGACTC	CRISPR/Cas9-mediated genome editing
mCasp3_gRNA_1_top	CACCGACTACTGCCGGAGTCTGAC	CRISPR/Cas9-mediated genome editing
mCasp3_gRNA_1_bottom	AAACGTCAGACTCCGGCAGTAGTC	CRISPR/Cas9-mediated genome editing
mCasp6_gRNA_1_top	CACCGTGAAGCAATCGGCATCTATG	CRISPR/Cas9-mediated genome editing
mCasp6_gRNA_1_bottom	AAACCATAGATGCCGATTGCTTCAC	CRISPR/Cas9-mediated genome editing
mCasp7_gRNA_1_top	CACCGGTCCCGGCCGGTCCTGACG	CRISPR/Cas9-mediated genome editing
mCasp7_gRNA_1_bottom	AAACCGTCAGGACCGGCCGGGACC	CRISPR/Cas9-mediated genome editing
mCasp8_gRNA_1_top	CACCGAGCCTGCTGGGGAAGATCG	CRISPR/Cas9-mediated genome editing
mCasp8_gRNA_1_bottom	AAACCGATCTTCCCCAGCAGGCTC	CRISPR/Cas9-mediated genome editing
mCasp9_gRNA_1_top	CACCGACCCGTCACAGCCTGCCGT	CRISPR/Cas9-mediated genome editing

mCasp9_gRNA_1_bottom	AAACACGGCAGGCTGTGACGGGTC	CRISPR/Cas9-mediated genome editing
mCasp9_gRNA_2_top	CACCGAAGTTTGTCACGGTCCAAGT	CRISPR/Cas9-mediated genome editing
mCasp9_gRNA_2_bottom	AAACACTTGGACCGTGACAAACTTC	CRISPR/Cas9-mediated genome editing
mRipk3_gRNA_1_top	CACCGAGGGTTCGGAGTCGTGTTC	CRISPR/Cas9-mediated genome editing
mRipk3_gRNA_1_bottom	AAACGAACACGACTCCGAACCCTC	CRISPR/Cas9-mediated genome editing
BamHI-SpeI-HA_top	CTAGCGGATCCGGAACTAGTTATCCGTACGACGTACCAGACTACGCATAAGATATCGTCGAC	Generation of CL26-iCaspases cells
BamHI-SpeI-HA_bottom	GATCGTCGACGATATCTTATGCGTAGTCTGGTACGTCGTACGGATAACTAGTTCCGGATCCG	Generation of CL26-iCaspases cells
mCasp1_FL_S	AGAAACGCCATGGCTGACAAG	Generation of CL26-iCaspases cells
mCasp1_FL_A	GGATTCTTCGTTTAATGTCC	Generation of CL26-iCaspases cells
mCasp8_FL_S	ATGGATTTCCAGAGTTGTCTT	Generation of CL26-iCaspases cells
mCasp8_FL_A	TCATTAGGGAGGGAAGAAGA	Generation of CL26-iCaspases cells
pLentiCas9T2ABFP_HpaI_A1	GATCCGTTAACCATGGTGGCAGCGCTCTAGAAC	Generation of pLenti-T2A-BFP
pLentiCas9T2ABFP_HpaI_S1	CCATGGTTAACGGATCCGGAGAGGGCAGAGGAA	Generation of pLenti-T2A-BFP
pLentiCas9T2ABFP_S2	ACAGAATCAGGGGATAACGCAGG	Generation of pLenti-T2A-BFP
pLentiCas9T2ABFP_A2	ATCCCCTGATTCTGTGGATAACC	Generation of pLenti-T2A-BFP
pLentiT2ABFP_HpaIFvCasp_S	GCTGCCACCATGGTTGCCGCCACCATGGCTTCTAG	Generation of L929-iCasp1 cells
pLentiT2ABFP_HpaIFvCasp_A	CTCTCCGGATCCGTTTGCGTAGTCTGGTACGTCGTAC	Generation of L929-iCasp1 cells
mGsdmd_qPCR_S	GCGATCTCATTCCGGTGGACAG	Quantitative RT-PCR
mGsdmd_qPCR_A	TTCCCATCGACGACATCAGAGAC	Quantitative RT-PCR
mGsdme_qPCR_S	CAGCTGGTGGGATACAGGATAC	Quantitative RT-PCR
mGsdme_qPCR_A	CTGTCATCAGACAGAGCATGGAG	Quantitative RT-PCR
mBax_qPCR_S1	TGAAGACAGGGGCCTTTTTG	Quantitative RT-PCR
mBax_qPCR_A1	AATTCGCCGGAGACACTCG	Quantitative RT-PCR
mBak1_qPCR_S1	CGTAGCGCCGGTTAATATCAT	Quantitative RT-PCR
mBak1_qPCR_A1	CGTAGCGCCGGTTAATATCAT	Quantitative RT-PCR
mGapdh_qPCR_S	TGACCACAGTCCATGCCATC	Quantitative RT-PCR
mGapdh_qPCR_A	GACGGACACATTGGGGGTAG	Quantitative RT-PCR
mGsdmd_FL_S	AGGTCCTCGCTTCGCTTGGTGGAC	Rescue experiments

mGsdmd_FL_A	AGGCTAACAAGGTTTCTGGCCTAGAC	Rescue experiments
M13F-97	TCGGTGCGGGCCTCTTCGCTATTAC	Rescue experiments
mGsdmd_DelSTOP_A	GGCCCGAATTCCCACAAGGTTTCTGGCCTAGACTT	Rescue experiments
mCasp9_T2ABFP_HpaI_S1,	GCTGCCACCATGGTTGACGAGGCGGACCGGCAGCTC	Rescue experiments
mCasp9_T2ABFP_HpaI_A1	CTCTCCGGATCCGTTTGAAGTTTTAAAAAACAGCTTTTTCC	Rescue experiments
pLSFFV_mGSDMDs_MluI	ATGCTACGCGTCCATCGGCCTTTGAGAAAGTG	Rescue experiments
pLSFFV_mGSDMDa_MluI	ATGCTACGCGTACAAGGTTTCTGGCCTAGACT	Rescue experiments
mGsdmd_I105N_S	GGGAAAAATTCTGGTGGGGCTGCAGT	Site-directed mutagenesis
mGsdmd_I105N_A	CACCAGAATTTTTCCCTTCTCCCATGC	Site-directed mutagenesis
mBid_D59A_S	CAGACAGCTGGCAGCCAGGCCAGCCG	Site-directed mutagenesis
mBid_D59A_A	GCTGCCAGCTGTCTGCAGCTCGTCTT	Site-directed mutagenesis
mBid_D75A_S	GAGCCAGCTTCTGAAAGTCAGGAAGA	Site-directed mutagenesis
mBid_D75A_A	TTCAGAAGCTGGCTCTATTCTTCCTT	Site-directed mutagenesis

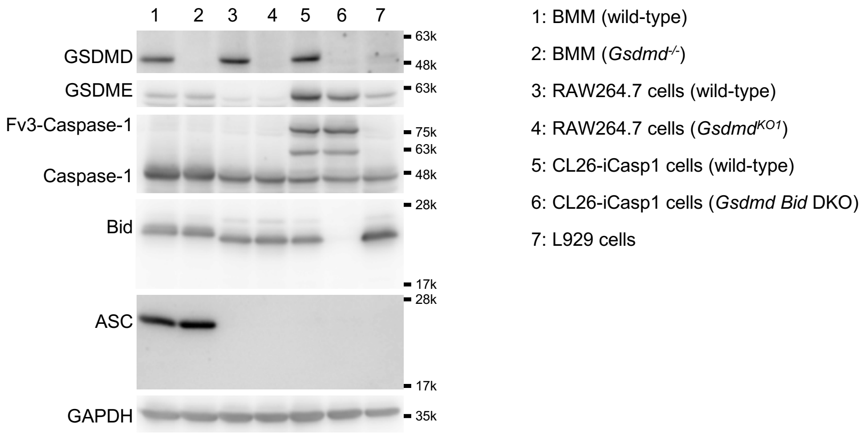

Supplementary Fig. 1 Protein levels of GSDMD, GSDME, caspase-1, Bid, and ASC in cell lines used in this study. Proteins were extracted from BMMs, RAW264.7 cells, CL26-iCasp1 cells, and L929 cells and subjected to Western blotting (20 μg protein per lane). Data are from one representative of two independent experiments with similar results.

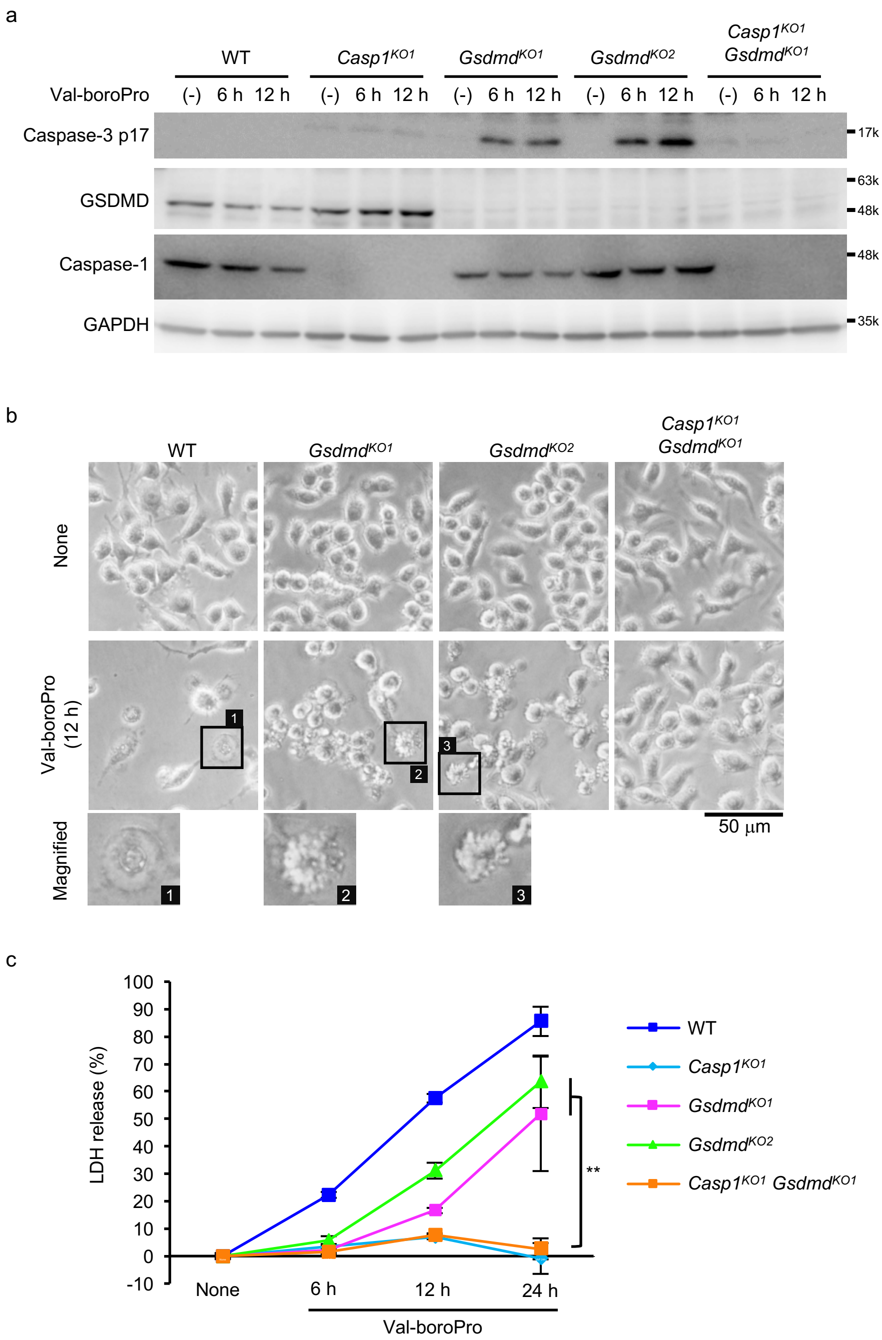

Supplementary Fig. 2 GSDMD-deficient RAW264.7 cells undergo apoptosis after Val-boroPro treatment. **a-c** RAW264.7 cells of the indicated genotypes were treated with 4 µM Val-boroPro for the indicated times. Cleaved caspase-3 in the cells was detected by Western blotting (a). Microscopic images of the cells (b). LDH release (c). Graph depicts the mean ± SD of triplicate cultures. Statistical significance was determined using Bonferroni's multiple comparisons test. **$p < 0.01$. Data are from one representative of three independent experiments with similar results (a-c). Source data are provided as a Source Data file.

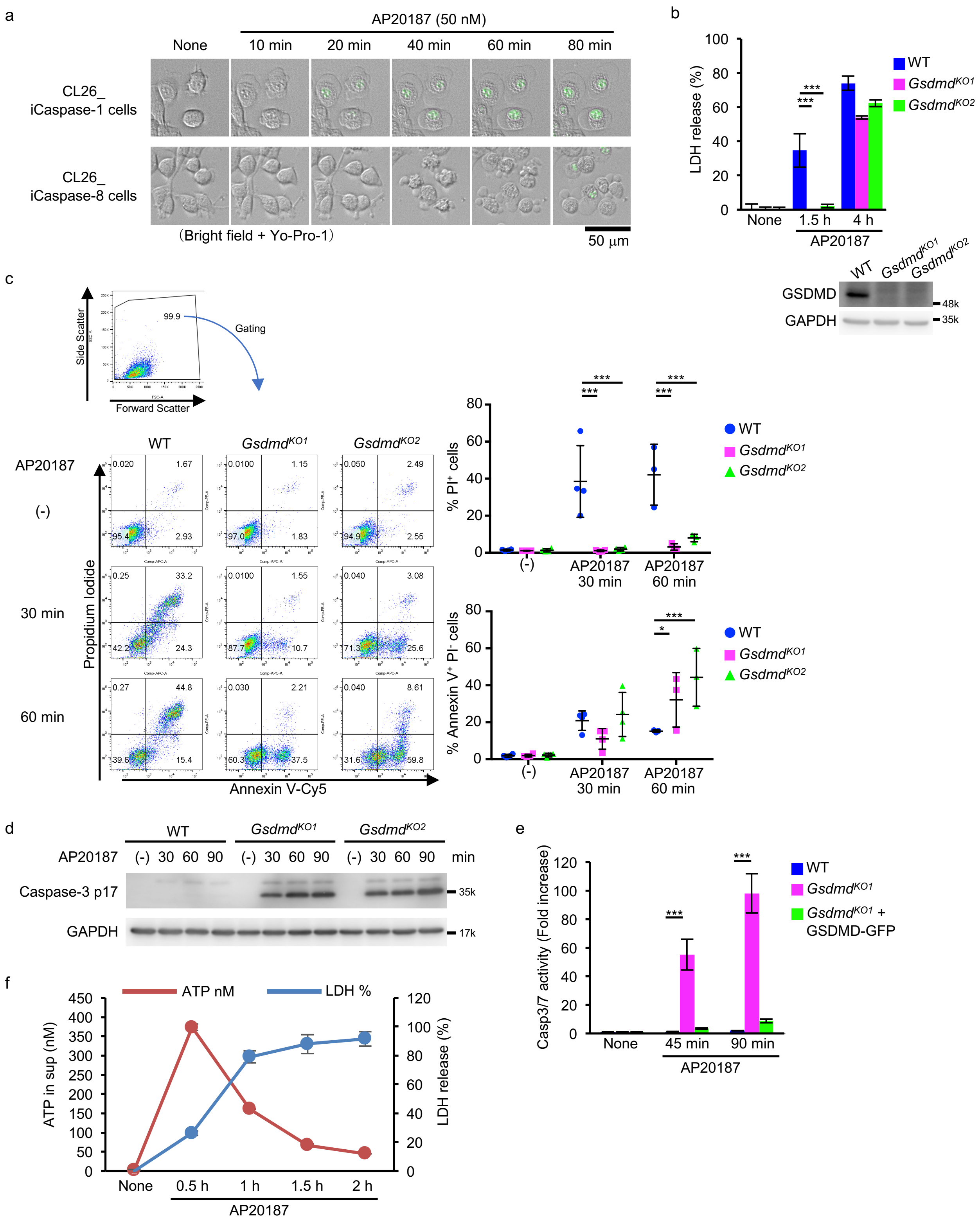

Supplementary Fig. 3 GSDMD-null cells undergo apoptosis after caspase-1 activation. **a** Time-lapse images of CL26-iCasp1 cells and CL26-iCasp8 cells after treatment with 50 nM AP20187 in the presence of Yo-Pro-1, a cell-impermeant fluorescent dye. Yo-Pro-1 fluorescence is shown in green. **b-d** WT and *Gsdmd*-KO CL26-iCasp1 cells were treated with or without AP20187 for the indicated times. LDH release was measured, and GSDMD in cell lysates was detected by Western blotting (b). PI uptake and PS exposure were analyzed by flow cytometry (c). Gating strategy, representative flow

cytometry profiles, and the graphs that show percentages of PI^+ (necrotic) cells and Annexin V^+ PI^- (early apoptotic) cells from four independent experiments (untreated and 30 min, $n = 4$; 60min, $n = 3$) are shown. Horizontal and vertical bars indicate the mean ± SD. Cleaved caspase-3 in culture supernatants plus cell lysates was detected by Western blotting (d). **e** Caspase-3/7 activity in cell lysates of AP20187-treated CL26-iCasp1 cell lines was determined using a proluminescent caspase-3/7 substrate. **f** WT CL26-iCasp1 cells were treated with AP20187, and the release of ATP and LDH was monitored. In b, e and f, graphs depict the mean ± SD of triplicate cultures. Data are from one representative of two (a,f) or three (b,d,e) independent experiments with similar results. Statistical significance was determined using Bonferroni's multiple comparisons test (b,c,e). $*p < 0.05$ and $***p < 0.001$. Source data are provided as a Source Data file.

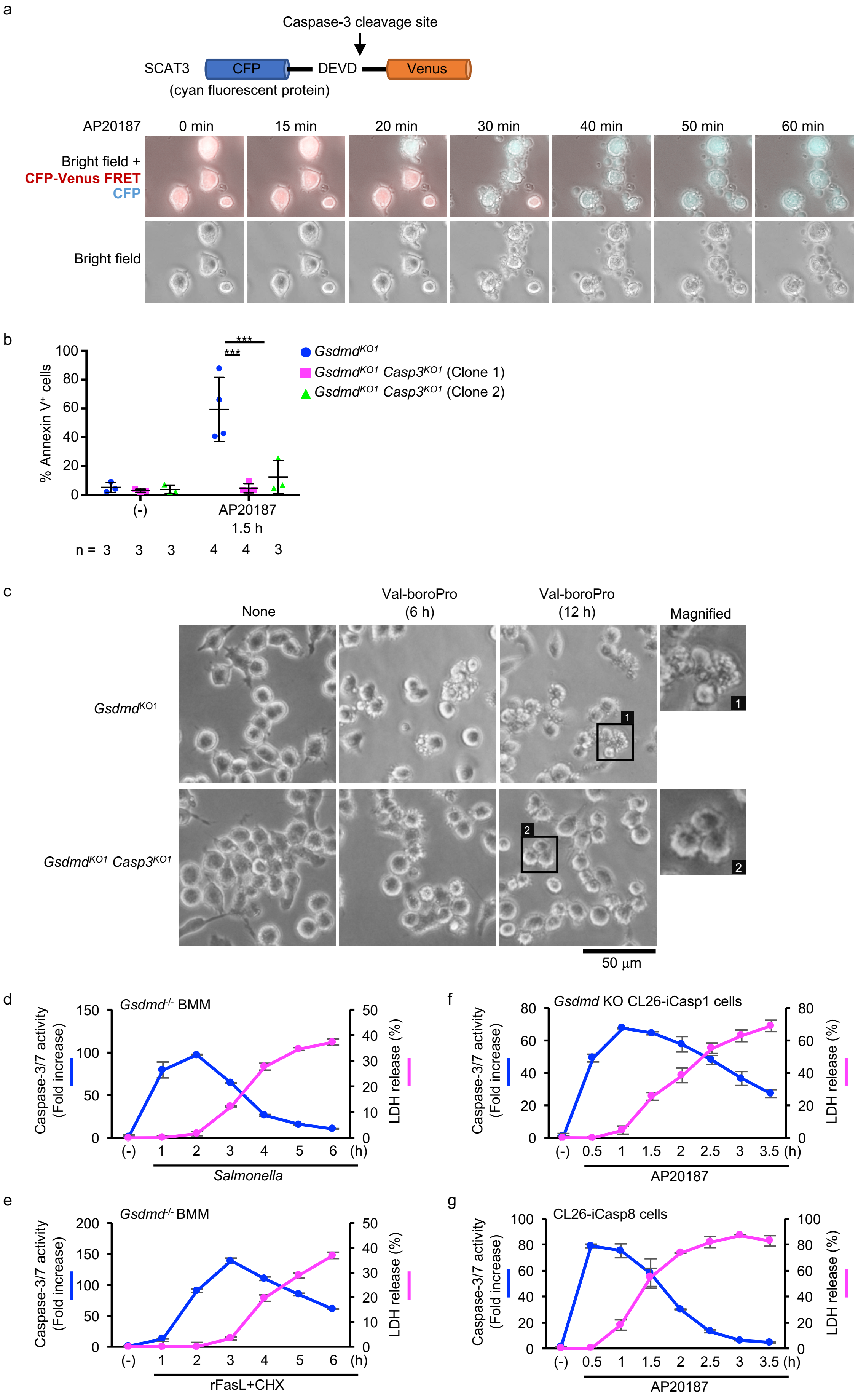
a
Caspase-3 cleavage site
SCAT3
CFP
DEVD
Venus
(cyan fluorescent protein)
AP20187
0 min
15 min
20 min
30 min
40 min
50 min
60 min
Bright field +
CFP-Venus FRET
CFP
Bright field
b
% Annexin V+ cells
100
80
60
40
20
0
***
***
Gsdmd^KO1
Gsdmd^KO1 Casp3^KO1 (Clone 1)
Gsdmd^KO1 Casp3^KO1 (Clone 2)
(-)
AP20187
1.5 h
n = 3 3 3 4 4 3
c
None
Val-boroPro (6 h)
Val-boroPro (12 h)
Magnified
Gsdmd^KO1
Gsdmd^KO1 Casp3^KO1
1
2
50 μm
d
Gsdmd-/- BMM
Caspase-3/7 activity (Fold increase)
LDH release (%)
(-) 1 2 3 4 5 6 (h)
Salmonella
e
Gsdmd-/- BMM
Caspase-3/7 activity (Fold increase)
LDH release (%)
(-) 1 2 3 4 5 6 (h)
rFasL+CHX
f
Gsdmd KO CL26-iCasp1 cells
Caspase-3/7 activity (Fold increase)
LDH release (%)
(-) 0.5 1 1.5 2 2.5 3 3.5 (h)
AP20187
g
CL26-iCasp8 cells
Caspase-3/7 activity (Fold increase)
LDH release (%)
(-) 0.5 1 1.5 2 2.5 3 3.5 (h)
AP20187

Supplementary Fig. 4 Caspase-1-induced apoptosis depends on caspase-3. **a** Time-lapse images of AP20187-treated *Gsdmd*-KO CL26-iCasp1 cells stably transfected with SCAT3, a fluorescence resonance energy transfer (FRET) probe used to detect caspase-3 activation. **b** Flow cytometric analysis of PI uptake and PS exposure in *Gsdmd*-KO and *Gsdmd*/*Casp3*-DKO CL26-iCasp1 cells treated with AP20187 as in Fig. 3c. Percentages of Annexin V^+ cells (n = 3-4 as shown at the bottom) from three independent experiments are plotted. Horizontal and vertical bars indicate the mean ± SD. Statistical significance was determined using Bonferroni's multiple comparisons test. ***$p < 0.001$. **c** *Gsdmd*-KO and *Gsdmd*/*Casp3*-DKO RAW264.7 cells were treated with Val-boro-Pro (4 µM), and microscopic images of the cells are shown. **d-g** *Gsdmd*$^{-/-}$ BMMs were infected with *Salmonella* at an MOI of 5 (d) or treated with 100 ng ml^{-1} of recombinant Fas ligand plus 1 µg ml^{-1} of cycloheximide (e). *Gsdmd* KO CL26-iCasp1 cells (f) and CL26-iCasp8 cells (g) were treated with AP20187. At the indicated time points, LDH release was measured, and Caspase-3/7 activity in cell lysates was determined (d-g). In g-f, graphs depict the mean ± SD of triplicate cultures. Data are from one representative of two (a,f,g) or three (c) independent experiments or two biologically independent experiments (d,e) with similar results. Source data are provided as a Source Data file.

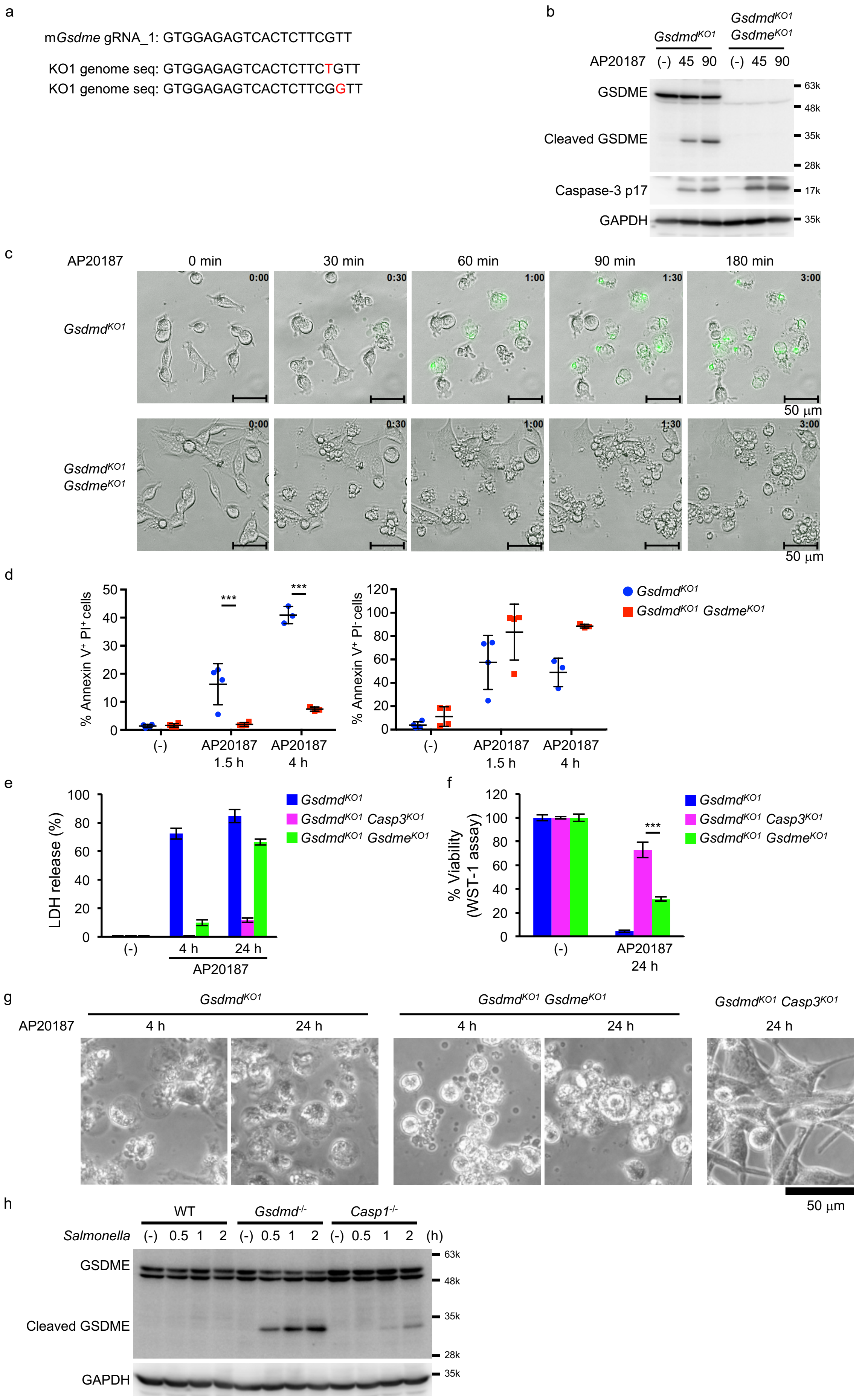
a
mGsdme gRNA_1: GTGGAGAGTCACTCTTCGTT
KO1 genome seq: GTGGAGAGTCACTCTTCTGTT
KO1 genome seq: GTGGAGAGTCACTCTTCGGTT
b
GsdmdKO1
GsdmdKO1 GsdmeKO1
AP20187 (-) 45 90 (-) 45 90
GSDME
Cleaved GSDME
Caspase-3 p17
GAPDH
c
AP20187 0 min 30 min 60 min 90 min 180 min
GsdmdKO1
GsdmdKO1 GsdmeKO1
50 μm
d
% Annexin V+ PI+ cells
% Annexin V+ PI- cells
(-) AP20187 1.5 h AP20187 4 h
GsdmdKO1
GsdmdKO1 GsdmeKO1
e
LDH release (%)
(-) 4 h 24 h AP20187
GsdmdKO1
GsdmdKO1 Casp3KO1
GsdmdKO1 GsdmeKO1
f
% Viability (WST-1 assay)
(-) AP20187 24 h
g
GsdmdKO1
GsdmdKO1 GsdmeKO1
GsdmdKO1 Casp3KO1
AP20187 4 h 24 h 4 h 24 h 24 h
50 μm
h
WT Gsdmd-/- Casp1-/-
Salmonella (-) 0.5 1 2 (-) 0.5 1 2 (-) 0.5 1 2 (h)
GSDME
Cleaved GSDME
GAPDH

Supplementary Fig. 5 Role for GSDME in caspase-1-induced apoptosis. **a** Genomic sequences of *Gsdmd*/*Gsdme*-DKO CL26-iCasp1 cells. **b-d** *Gsdmd*-KO and *Gsdmd*/*Gsdme*-DKO CL26-iCasp1 cells were treated with AP20187. GSDME and cleaved caspase-3 were detected by Western blotting (b). Time-lapse images of the cells in the presence of Yo-Pro-1 (c). Yo-Pro-1 fluorescence is shown in green. Flow cytometric analysis of PI uptake and PS exposure in cells treated with AP20187 as in Fig. 3g (d). Percentages of Annexin V^+ PI^+ and Annexin V^+ PI^- cells (untreated and 1.5 h, $n = 4$; 4 h, $n = 3$) from three independent experiments are plotted. Horizontal and vertical bars indicate the mean ± SD. **e-g** *Gsdmd*-KO, *Gsdmd*/*Casp3*-DKO, and *Gsdmd*/*Gsdme*-DKO CL26-iCasp1 cells were treated with AP20187 for the indicated times. LDH release (e). Cell viability by WST-1 assay (f). Microscopic images (g). In e and f, graphs depict the mean ± SD of triplicate cultures. **h** BMMs of the indicated genotypes were infected with *S.* Typhimurium as in Fig. 1b. GSDME in culture supernatants plus cell lysates was detected by Western blotting. In d-f, statistical significance was determined using Bonferroni's multiple comparisons test. ***$p < 0.001$. In b, c, and e-h, data are from one representative of two (c) or three (b, e-h) independent experiments with similar results. Source data are provided as a Source Data file.

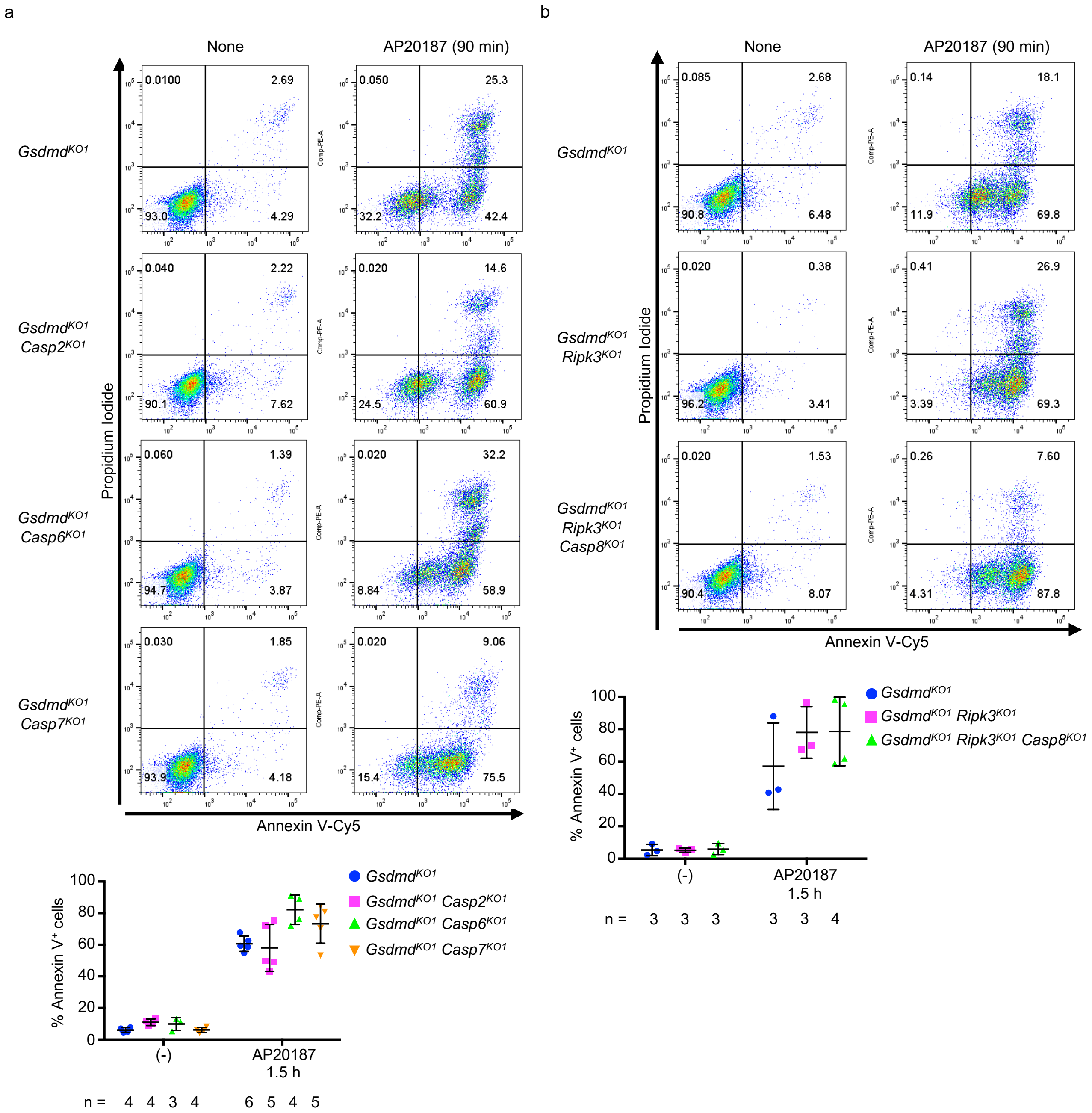

Supplementary Fig. 6 Caspase-2, -6, -7, and -8 are dispensable for the caspase-1-induced PS externalization. **a,b** CL26-iCasp1 cells of the indicated genotypes were treated with 50 nM AP20187 for 1.5 h. PI uptake and PS exposure of the cells were analyzed by flow cytometry. Representative flow cytometry profiles and the graphs that indicate percentages of Annexin V^+ cells (n = 3 - 6 as shown beneath the graphs) from two (b) or three (a) independent experiments are shown. Horizontal and vertical bars indicate the mean ± SD. Source data are provided as a Source Data file.

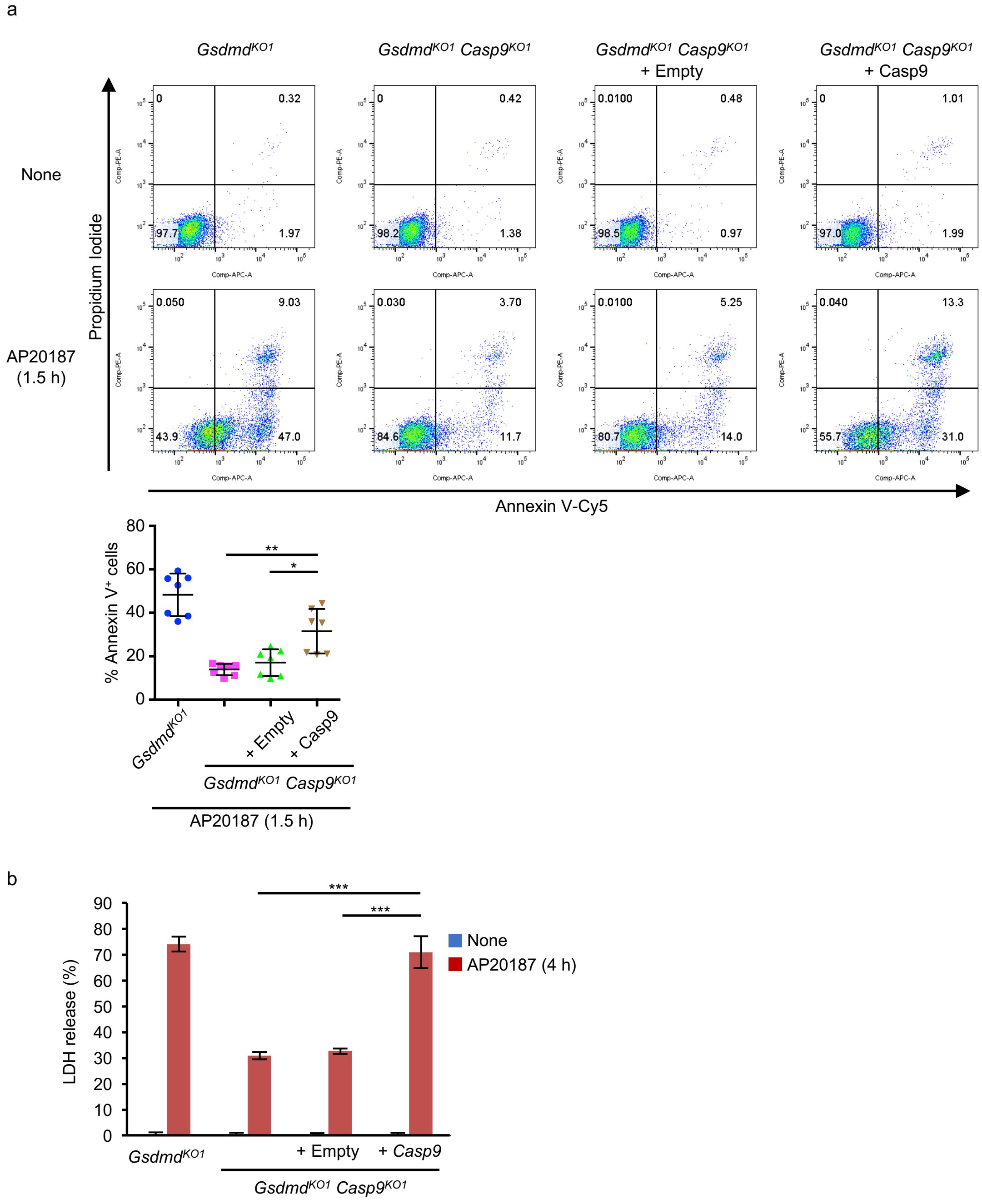

Supplementary Fig. 7 Expression of caspase-9 restores caspase-1-induced apoptosis in *Gsdmd*/*Casp9*-DKO cells. **a,b** *Gsdmd*-KO and *Gsdmd*/*Casp9*-DKO CL26-iCasp1 cells transduced or not transduced with Casp9 or empty vector control were treated with AP20187. Flow cytometric analysis of PI uptake and PS exposure (a). Representative flow cytometry profiles and the graph that shows percentages of Annexin V^+ cells (n = 7) from three independent experiments are shown. Horizontal and vertical bars indicate the mean ± SD. LDH release (b). The graph depicts the mean ± SD of triplicate cultures. Data are from one representative of three independent experiments with similar results. In a and b, statistical significance was determined using Bonferroni's multiple comparisons test. *p < 0.05, **p < 0.01, and ***p < 0.001. Source data are provided as a Source Data file.

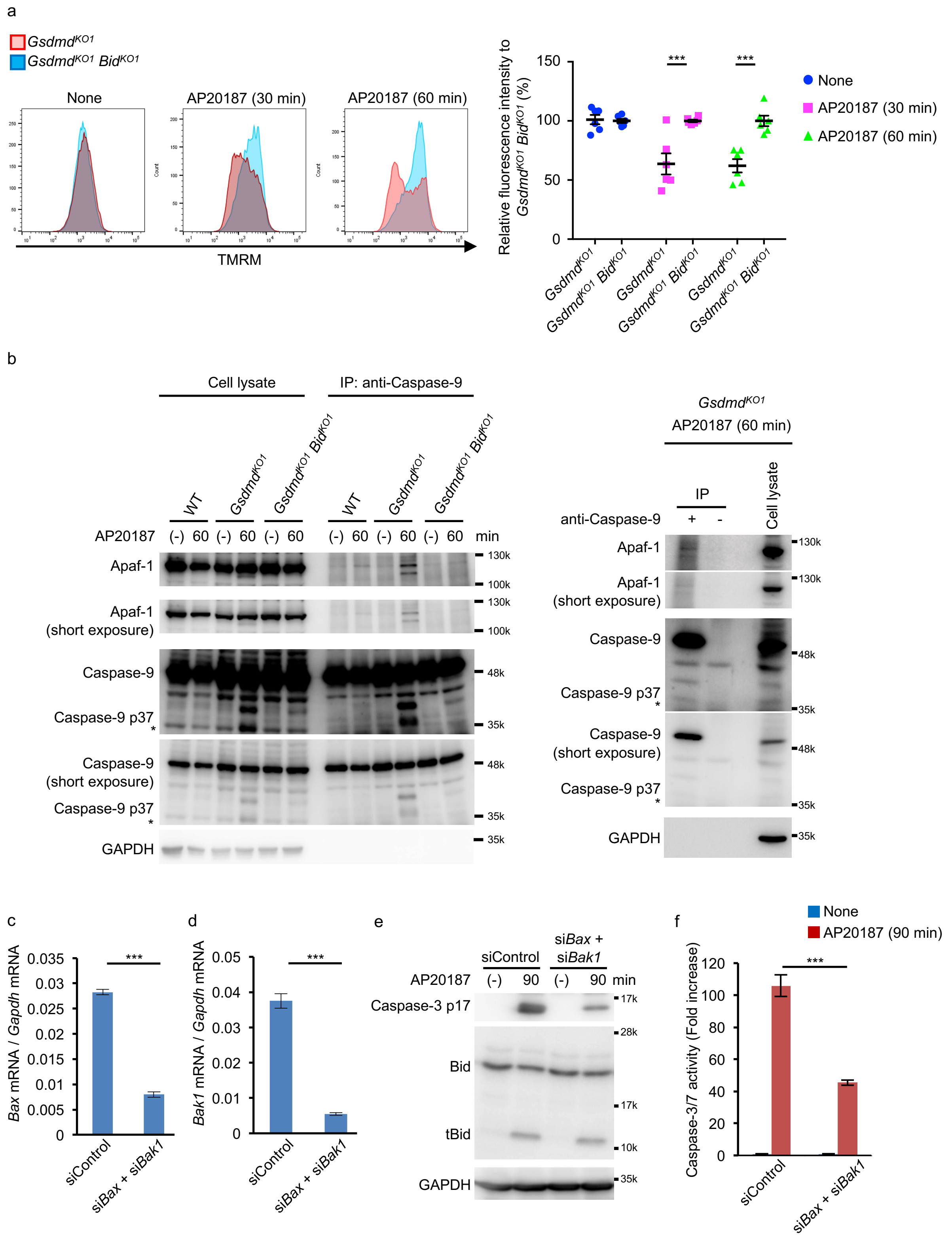

Supplementary Fig. 8 Mitochondrial depolarization and apoptosome formation during caspase-1-induced apoptosis. **a** *Gsdmd*-KO and *Gsdmd*/*Bid*-DKO CL26-iCasp1 cells left untreated or treated with AP20187 for the indicated times were stained with TMRM and analyzed by flow cytometry. The graph shows relative fluorescence intensity of cells to that of *Gsdmd*/*Bid*-DKO cells cultured under the same conditions from three independent experiments (n = 6). Horizontal and vertical bars indicate the mean ± SD. **b** CL26-iCasp1 cells of the indicated genotypes were treated with or without

AP20187 for 1 h. Whole cell lysates and immunoprecipitates with or without anti-caspase-9 antibody were subjected to Western blotting for Apaf-1 and caspase-9. **c-f** *Gsdmd*-KO CL26-iCasp1 cells were transfected with control siRNA or *Bax* siRNA plus *Bak1* siRNA. Two days after transfection, the cells were again transfected with the same siRNAs and incubated for an additional 2 days. Cells were then treated with or without AP20187. Quantitative RT-PCR analysis of Bax (c) and Bak1 (d) mRNA expression without AP20187 treatment. Cleaved caspase-3 and Bid in cell lysates were detected by Western blotting (e). Caspase-3/7 activity in the cell lysates (f). In c, d and f, graphs depict the mean ± SD of triplicate cultures. In a, c, d and f, statistical significance was determined using an unpaired Student's t-test. ***$p < 0.001$. Data are from one representative of three independent experiments with similar results (b-f). Source data are provided as a Source Data file.

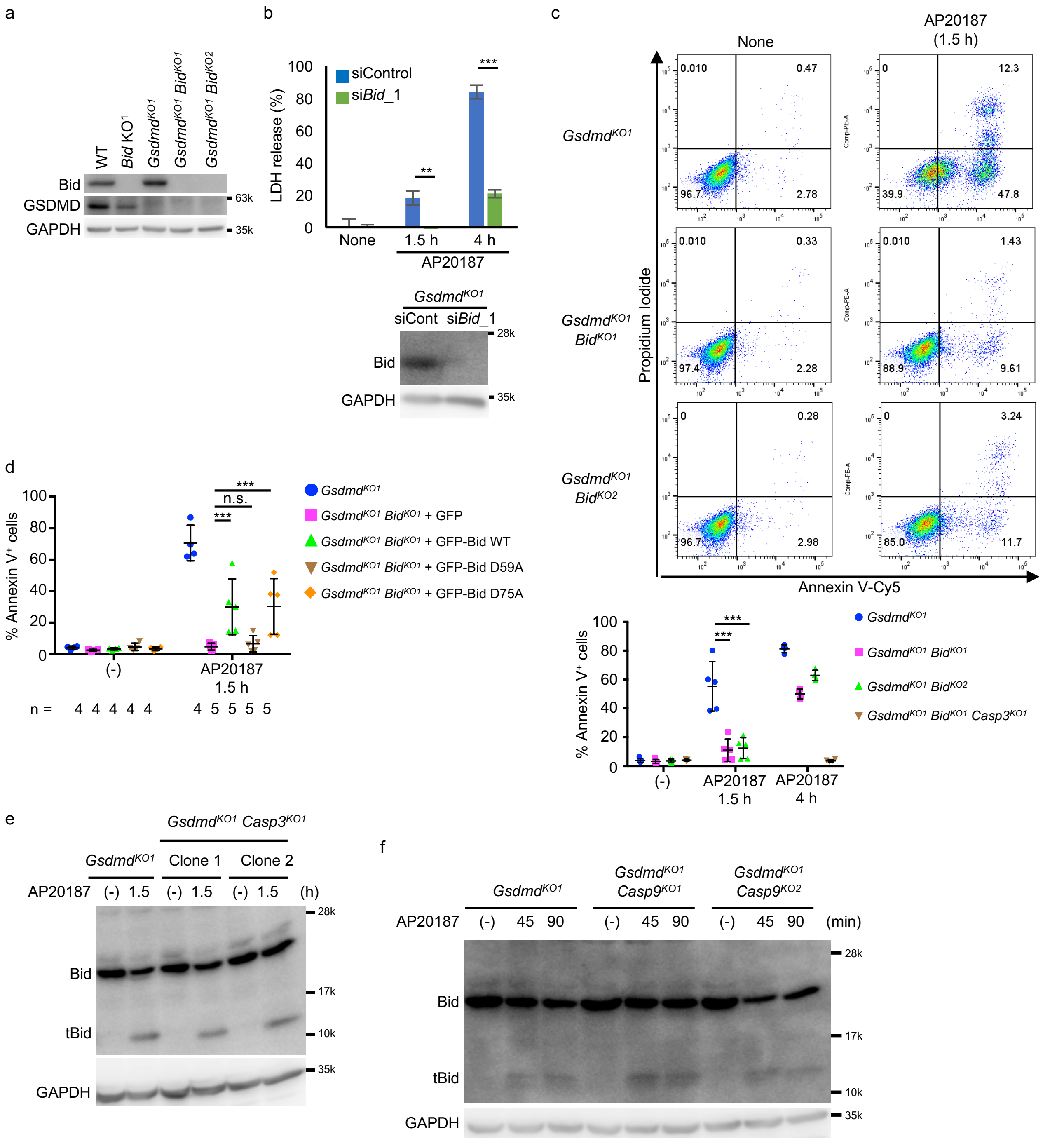

Supplementary Fig. 9 Role of Bid in caspase-1-induced apoptosis. **a** Western blot detection of Bid and GSDMD in CL26-iCasp1 cells of the indicated genotypes. **b** *Gsdmd*-KO CL26-iCasp1 cells were transfected with control siRNA or *Bid* siRNA. Two days after transfection, the cells were treated with AP20187 for the indicated times, and cell death was monitored by LDH release assay (upper panel). The graph depicts the mean ± SD of triplicate cultures. Western blot detection of Bid in *Gsdmd*-KO CL26-iCasp1 cells transfected with control siRNA or *Bid* siRNA (lower panel). **c** Flow cytometric analysis of PI uptake and PS exposure. Representative flow cytometry profiles and the graph that shows percentages of Annexin V^+ cells from three independent experiments (untreated and 1.5 h, $n = 5$; 4 h, $n = 4$) are shown. **d** Flow cytometric analysis of PI uptake and PS exposure in cells treated with AP20187 as in Fig. 6g. Percentages of Annexin V^+ cells from three independent experiments are plotted (n = 4-5 as shown beneath the graph). In c and d, horizontal and vertical bars in the graphs

indicate the mean ± SD. In b-d, statistical significance was determined using Bonferroni's multiple comparisons test. n.s. not significant; $**p < 0.01$; $***p < 0.001$. **e,f** Western blot detection of Bid in CL26-iCasp1 cells of the indicated genotypes. In a, b, e and f, data are from one representative of three independent experiments with similar results. Source data are provided as a Source Data file.

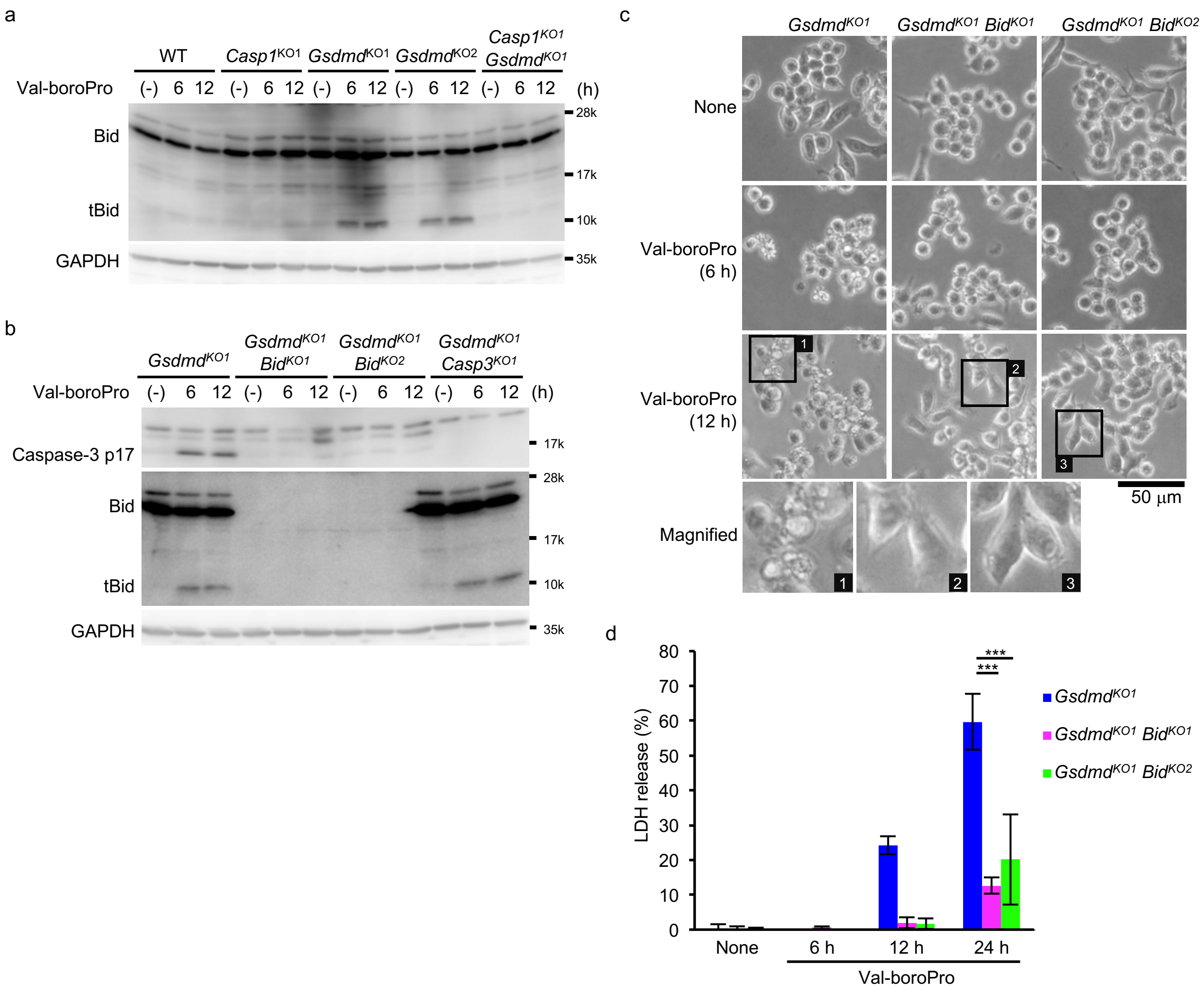

Supplementary Fig. 10 Involvement of Bid in caspase-1-induced apoptosis in macrophages. **a-d** RAW264.7 cells of the indicated genotypes were treated with 4 μM Val-boroPro for the indicated times. Bid and cleaved caspase-3 were detected by Western blotting (a,b). Microscopic images of the cells (c). LDH release (d). Graph depicts the mean ± SD of triplicate cultures (d). Statistical significance was determined using Bonferroni's multiple comparisons test. $***p < 0.001$. Data are from one representative of three independent experiments with similar results (a-d). Source data are provided as a Source Data file.

a

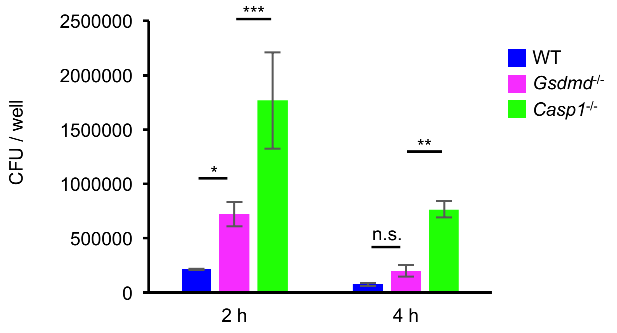

b

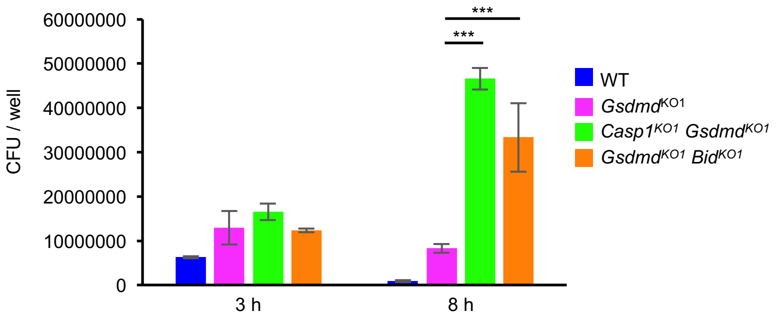

Supplementary Fig. 11 Effect of caspase-1-induced apoptosis on *S.* Typhimurium growth. **a,b** BMMs (a) and RAW264.7 cells (b) were infected with *S.* Typhimurium as in Fig. 1c (a) and Fig. 1e (b), respectively. The number of bacteria in the cells was assessed at the indicated times after infection. Graphs depict the mean ± SD of triplicate cultures. Statistical significance was determined using Bonferroni's multiple comparisons test (a) and an unpaired Student's t-test (b). n.s. not significant; $*p < 0.05$, $**p < 0.01$, and $***p < 0.001$. Data are from one representative of three independent experiments with similar results. Source data are provided as a Source Data file.

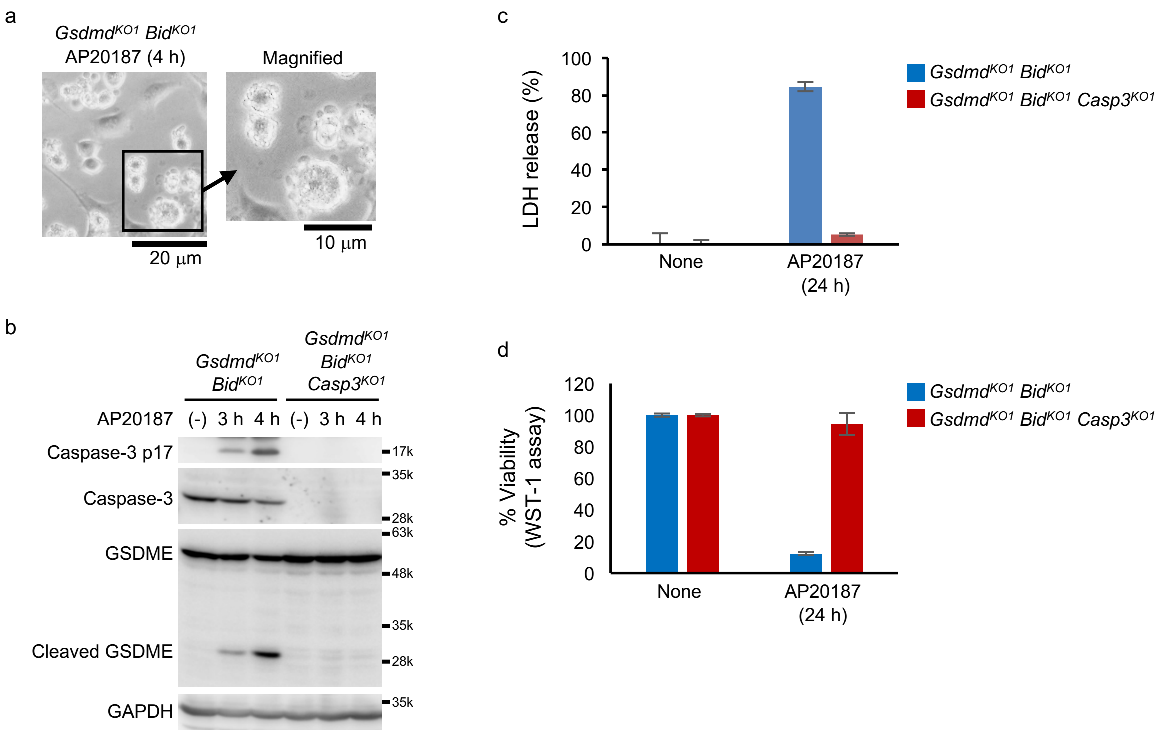

Supplementary Fig. 12 A bid-independent pathway depends on caspase-3. **a-c** *Gsdmd*/*Bid*-DKO and *Gsdmd*/*Bid*/*Casp3*-triple KO CL26-iCasp1 cells were treated with AP20187 for the indicated times. Microscopic images of *Gsdmd*/*Bid*-DKO cells treated with AP20187 for 4 h (a). Caspase-3 and GSDME were detected by Western blotting (b). LDH release (c). Cell viability by WST-1 assay (d). In c and d, graph depicts the mean ± SD of triplicate cultures. Data are from one representative of three independent experiments with similar results. Source data are provided as a Source Data file.

a

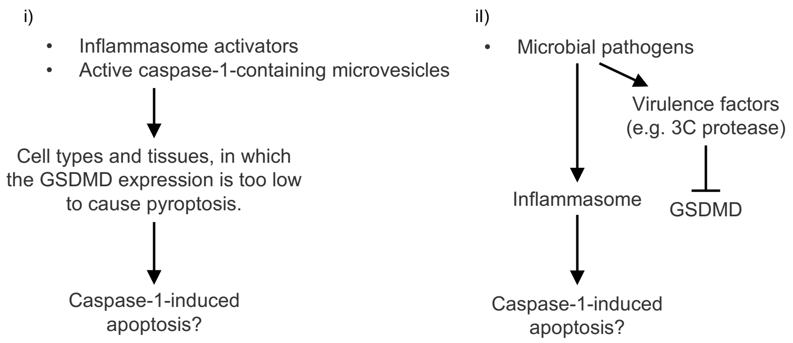

b

GeneAtlas MOE430, gcrma

Gene	*Gsdmd*	*Casp1*
Probe set	1428767_at	1449265_at
Number of sample	191	191
Median	81.7	66.8

Samples expressing GSDMD mRNA at high levels (Top 10)

Samples	*Gsdmd* (1428767_at)
Intestine_small.1	2239.351
Macrophage_bone_marrow_6hr_LPS.2	2065.621
Macrophage_bone_marrow_6hr_LPS.1	2018.945
Macrophage_peri_LPS_thio_7hrs.1	1941.688
Macrophage_peri_LPS_thio_7hrs.2	1838.528
Intestine_small.2	1700.205
RAW_264_7.1	1107.425
Macrophage_bone_marrow_24h_LPS.2	1104.834
Macrophage_bone_marrow_24h_LPS.1	1018.774
RAW_264_7.2	971.716

Selected samples

Selection criteria	Less than the median	Greater than twice the median
Samples	*Gsdmd* (1428767_at)	*Casp1* (1449265_at)
Epidermis.1	8.113	219.389
Mammary_gland__lact.1	15.46	301.348
Epidermis.2	26.018	488.879
Mammary_gland__lact.2	42.755	304.67
Mast_cells_IgE.1	45.904	343.443
Mast_cells_IgE.2	46.101	356.218
Mast_cells_IgE+antigen_1hr.1	64.011	272.33
Mast_cells_IgE+antigen_6hr.2	66.271	207.239
Mast_cells_IgE+antigen_6hr.1	66.867	275.077
B-cells_marginal_zone.1	74.941	770.929
B-cells_marginal_zone.2	79.117	724.311

c

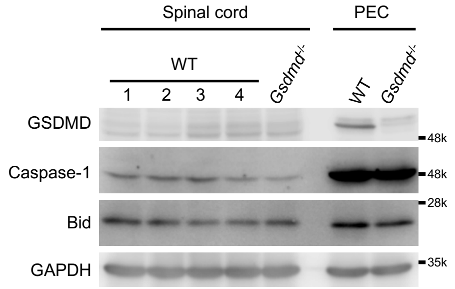

d

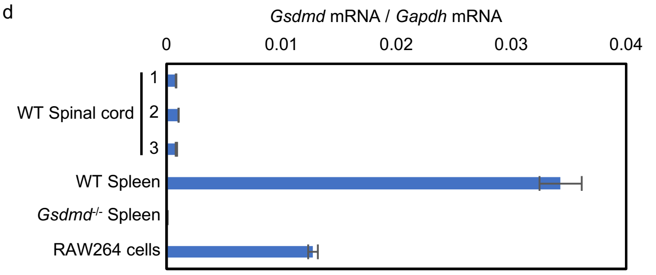

e

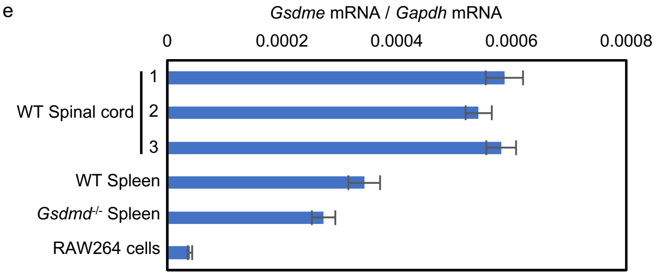

f

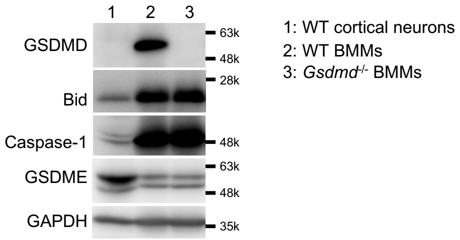

g

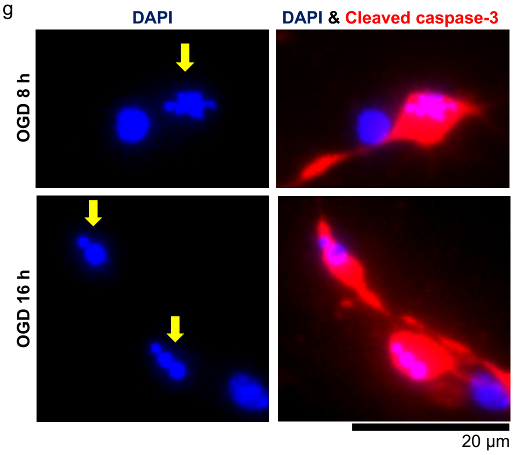

Supplementary Fig. 13 Caspase-1-induced apoptosis may occur in certain cell types. **a** Speculative situations in which caspase-1 may induce apoptosis. **b** Gene expression data [Dataset: GNF Mouse GeneAtlas V3 (GeneAtlas MOE430, gcrma; https://www.ncbi.nlm.nih.gov/geo/query/acc.cgi?acc=GSE10246); Probes: 1428767_at (*Gsdmd*), 1449265_at (*Casp1*)] were analyzed using BioGPS (http://biogps.org/#goto=welcome, top). Samples that express GSDMD mRNA at high levels (top

10) are listed (middle). Samples in which GSDMD mRNA levels and caspase-1 mRNA levels are relatively low and high, respectively, are listed according to the indicated criteria (bottom). **c** Western blot detection of GSDMD, Bid, and caspase-1 in the spinal cord and TEPMs from WT and *Gsdmd*$^{-/-}$ mice (21.4 μg protein per lane). **d,e** Quantitative RT-PCR analysis of GSDMD and GSDME mRNA expression in the spinal cord and spleen from WT and *Gsdmd*$^{-/-}$ mice and RAW264.7 cells. Each expression was calculated relative to GAPDH mRNA (the ΔCt method). **f** Western blot detection of GSDMs, Bid, and caspase-1 in primary cortical neurons and BMMs (14.3 μg protein per lane). **g** WT cortical neurons were treated with 8 and 16 h of OGD and processed for immunofluorescence staining with anti-cleaved caspase-3 (red) and DAPI (blue). Allows indicate apoptotic nuclei. Graphs depict the mean ± SD of technical triplicates (d,e). Data are from one representative of two (c-f) or three (g) biologically independent experiments with similar results: total 6 (c) or 5 (d,e) independent spinal cord samples from WT mice were examined. Source data are provided as a Source Data file.

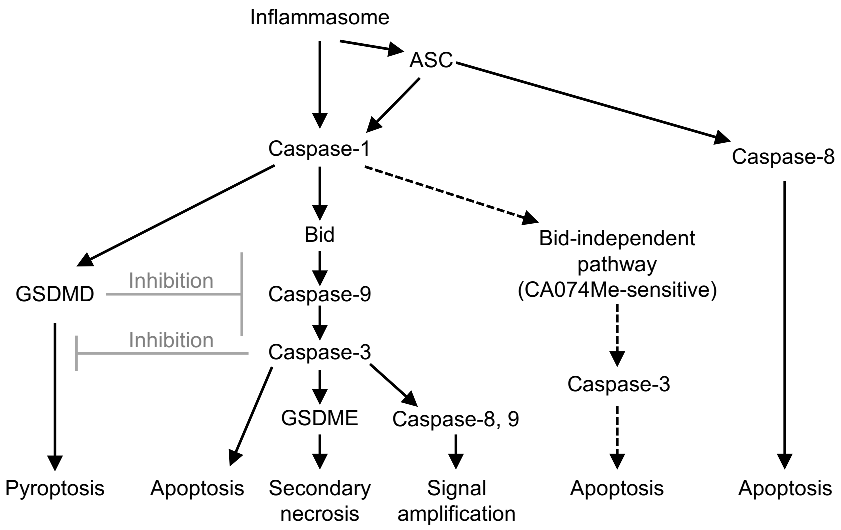

Supplementary Fig. 14 Proposed model for inflammasome-induced cell death cascades. In the presence of sufficient GSDMD, caspase-1 triggers pyroptosis. On the other hand, in the absence of GSDMD, inflammasomes induce apoptosis through at least three different pathways: the caspase-1-induced bid-dependent pathway, the caspase-1-induced bid-independent pathway, and the ASC/caspase-8-dependent pathway. Note that caspase-8 can also be activated downstream of caspase-1.

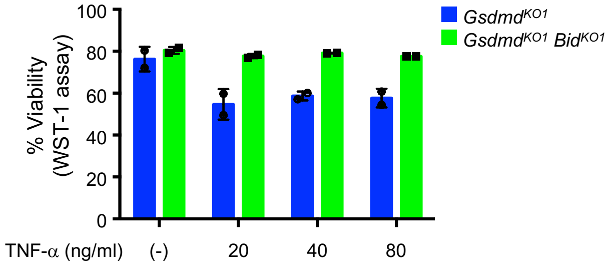

Supplementary Fig. 15 Bid is involved in tumor necrosis factor (TNF)-induced apoptosis in CL26-iCasp1 cells. *Gsdmd*-KO and *Gsdmd*/*Bid*-DKO CL26-iCasp1 cells were pretreated with cycloheximide (10 μg ml^{-1}) for 1 h and then treated with the indicated concentrations of TNF-α for 7 h. Cell viability was assessed by WST-1 assay. Viability of cells cultured without cycloheximide and TNF-α is set as 100%. Graph depicts the mean ± SD of duplicate cultures, and individual data values are plotted. Data are from one representative of two independent experiments with similar results. Source data are provided as a Source Data file.

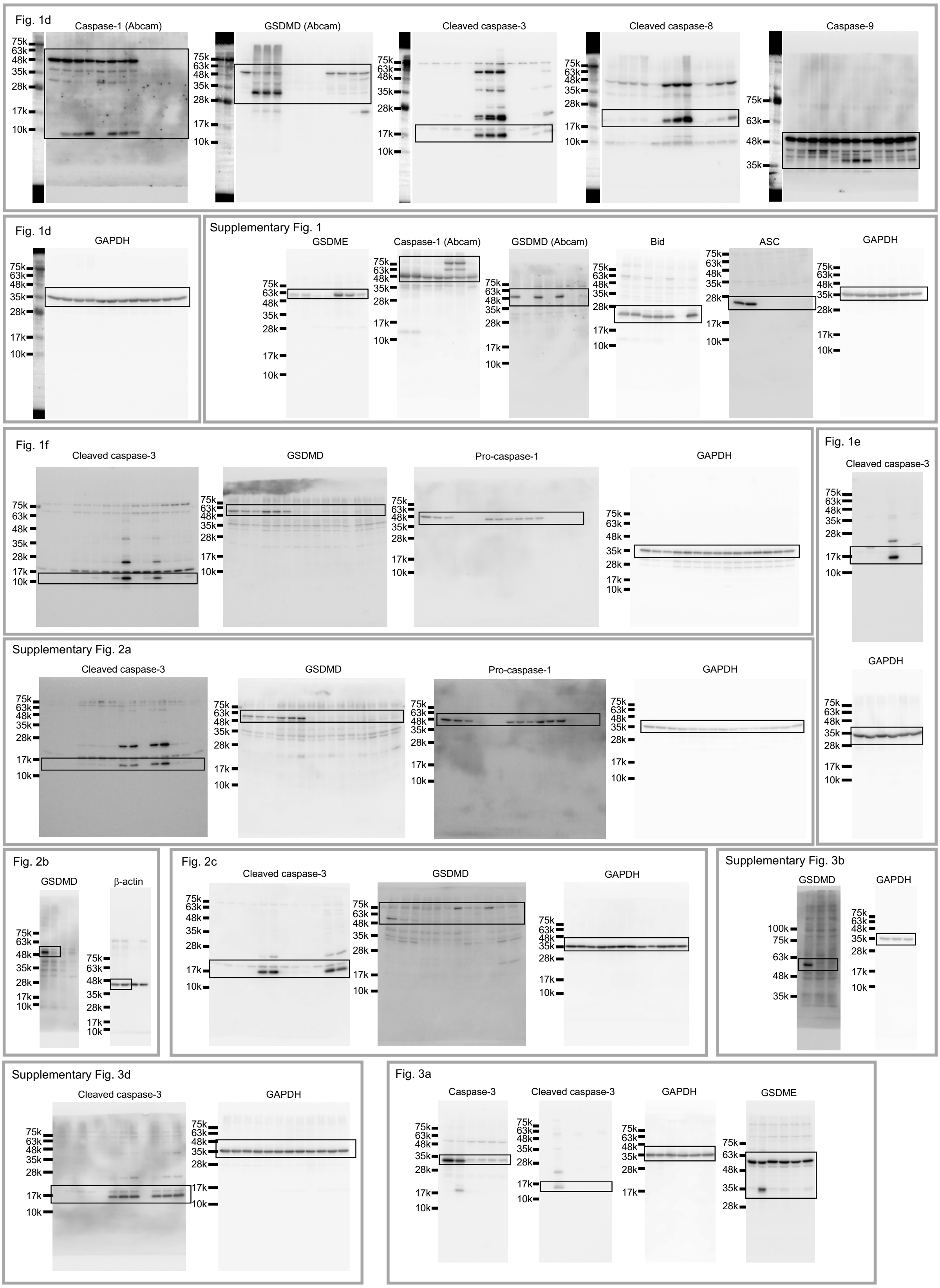
Fig. 1d
Caspase-1 (Abcam)
GSDMD (Abcam)
Cleaved caspase-3
Cleaved caspase-8
Caspase-9
Fig. 1d
GAPDH
Supplementary Fig. 1
GSDME
Caspase-1 (Abcam)
GSDMD (Abcam)
Bid
ASC
GAPDH
Fig. 1f
Cleaved caspase-3
GSDMD
Pro-caspase-1
GAPDH
Fig. 1e
Cleaved caspase-3
GAPDH
Supplementary Fig. 2a
Cleaved caspase-3
GSDMD
Pro-caspase-1
GAPDH
Fig. 2b
GSDMD
β-actin
Fig. 2c
Cleaved caspase-3
GSDMD
GAPDH
Supplementary Fig. 3b
GSDMD
GAPDH
Supplementary Fig. 3d
Cleaved caspase-3
GAPDH
Fig. 3a
Caspase-3
Cleaved caspase-3
GAPDH
GSDME
75k
63k
48k
35k
28k
17k
10k
100k

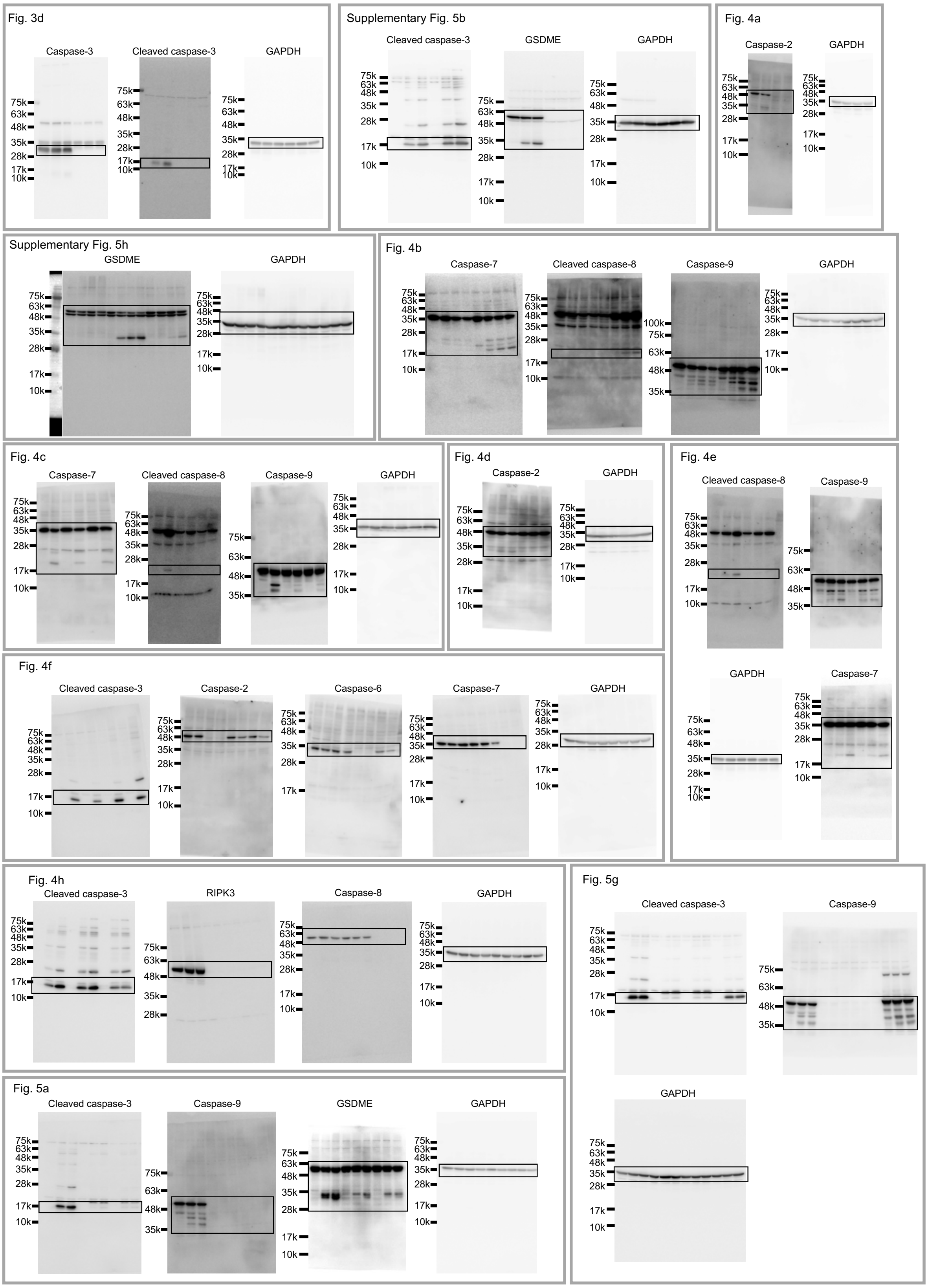
Fig. 3d
Caspase-3
Cleaved caspase-3
GAPDH
Supplementary Fig. 5b
Cleaved caspase-3
GSDME
GAPDH
Fig. 4a
Caspase-2
GAPDH
Supplementary Fig. 5h
GSDME
GAPDH
Fig. 4b
Caspase-7
Cleaved caspase-8
Caspase-9
GAPDH
Fig. 4c
Caspase-7
Cleaved caspase-8
Caspase-9
GAPDH
Fig. 4d
Caspase-2
GAPDH
Fig. 4e
Cleaved caspase-8
Caspase-9
GAPDH
Caspase-7
Fig. 4f
Cleaved caspase-3
Caspase-2
Caspase-6
Caspase-7
GAPDH
Fig. 4h
Cleaved caspase-3
RIPK3
Caspase-8
GAPDH
Fig. 5g
Cleaved caspase-3
Caspase-9
GAPDH
Fig. 5a
Cleaved caspase-3
Caspase-9
GSDME
GAPDH

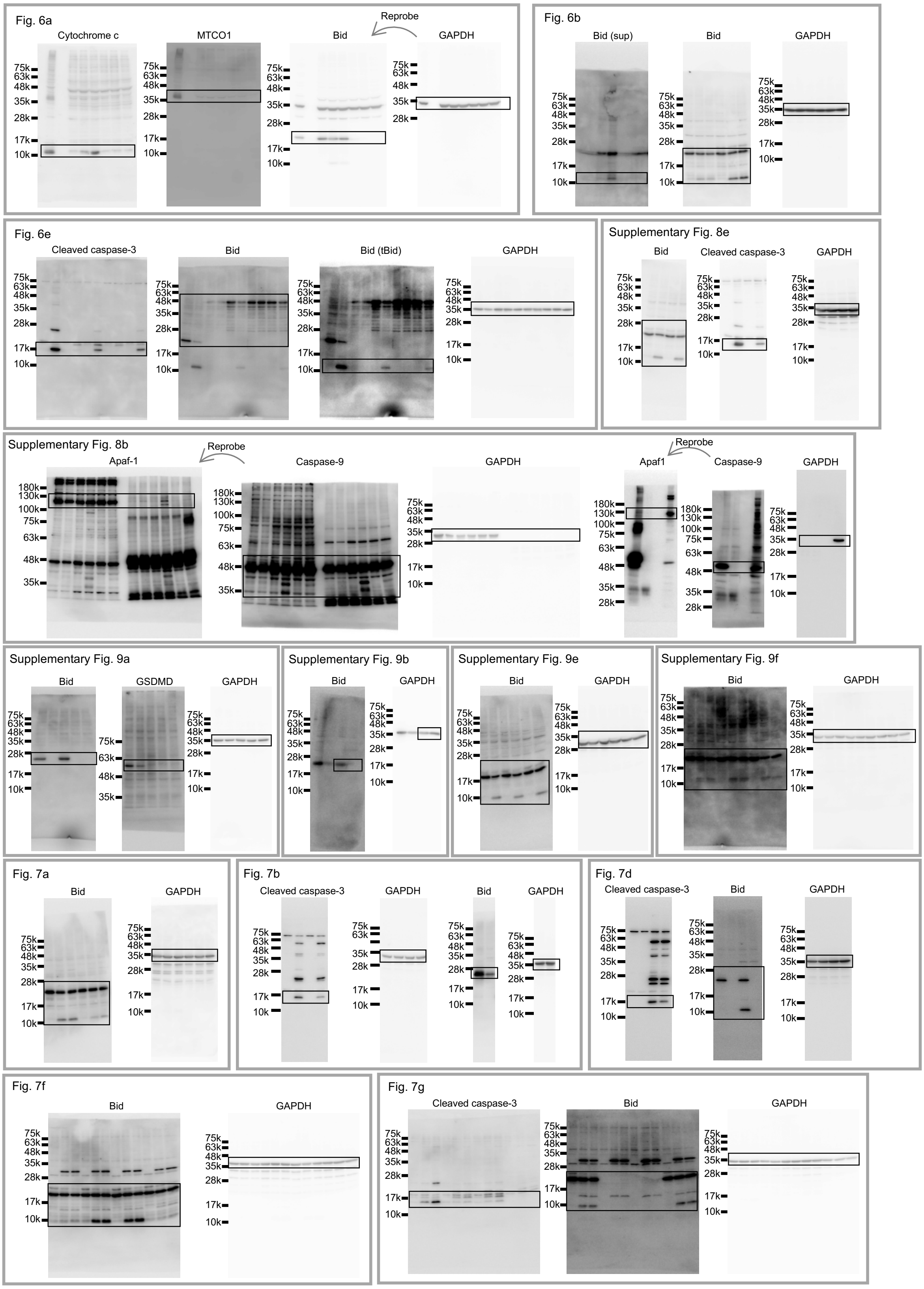

Fig. 6a
Cytochrome c
MTCO1
Bid
Reprobe
GAPDH
Fig. 6b
Bid (sup)
Bid
GAPDH
Fig. 6e
Cleaved caspase-3
Bid
Bid (tBid)
GAPDH
Supplementary Fig. 8e
Bid
Cleaved caspase-3
GAPDH
Supplementary Fig. 8b
Apaf-1
Reprobe
Caspase-9
GAPDH
Reprobe
Apaf1
Caspase-9
GAPDH
Supplementary Fig. 9a
Bid
GSDMD
GAPDH
Supplementary Fig. 9b
Bid
GAPDH
Supplementary Fig. 9e
Bid
GAPDH
Supplementary Fig. 9f
Bid
GAPDH
Fig. 7a
Bid
GAPDH
Fig. 7b
Cleaved caspase-3
GAPDH
Bid
GAPDH
Fig. 7d
Cleaved caspase-3
Bid
GAPDH
Fig. 7f
Bid
GAPDH
Fig. 7g
Cleaved caspase-3
Bid
GAPDH

Supplementary Fig. 10a

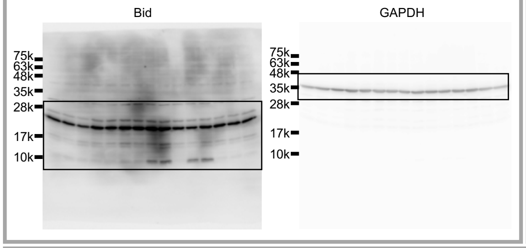

Supplementary Fig. 10b

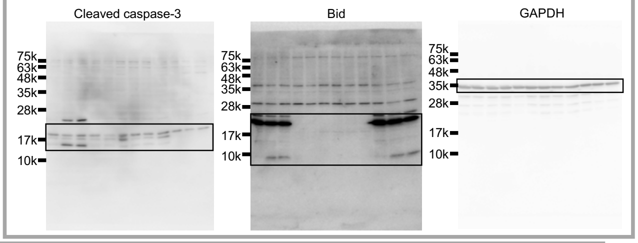

Fig. 8a

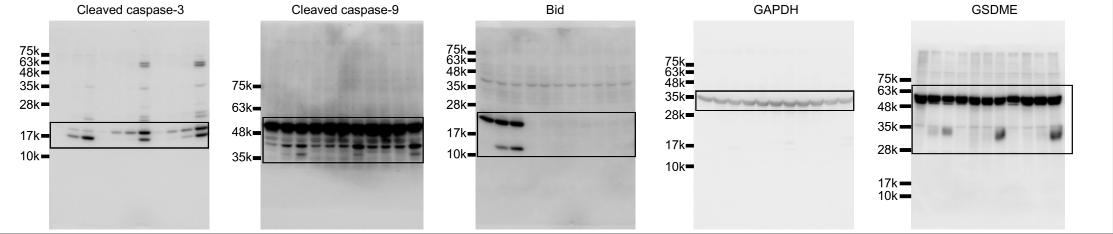

Fig. 8g

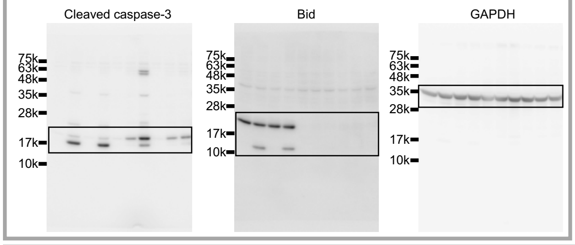

Supplementary Fig. 12b

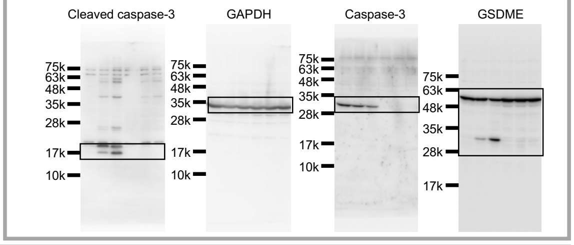

Fig. 9a

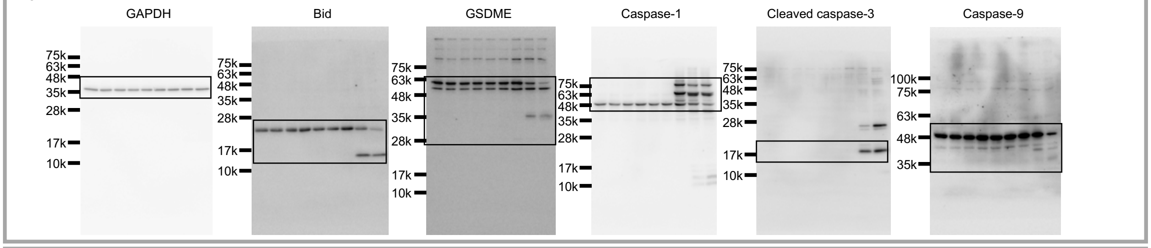

Fig. 9d

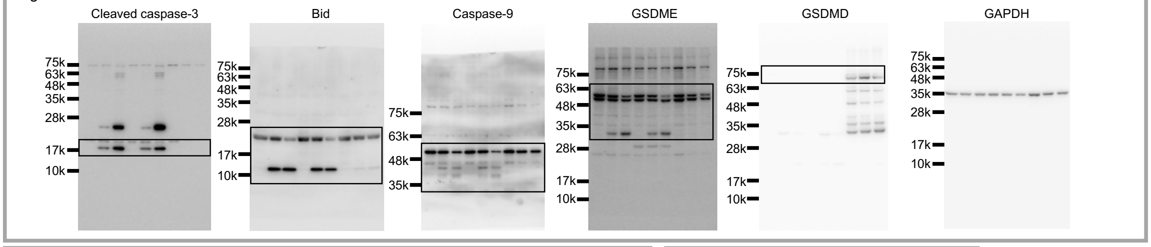

Supplementary Fig. 13c

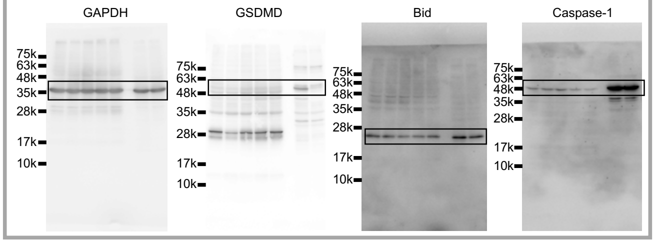

Fig. 10f

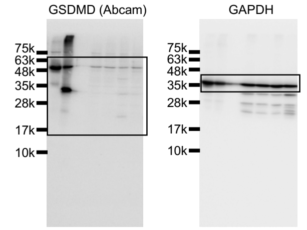

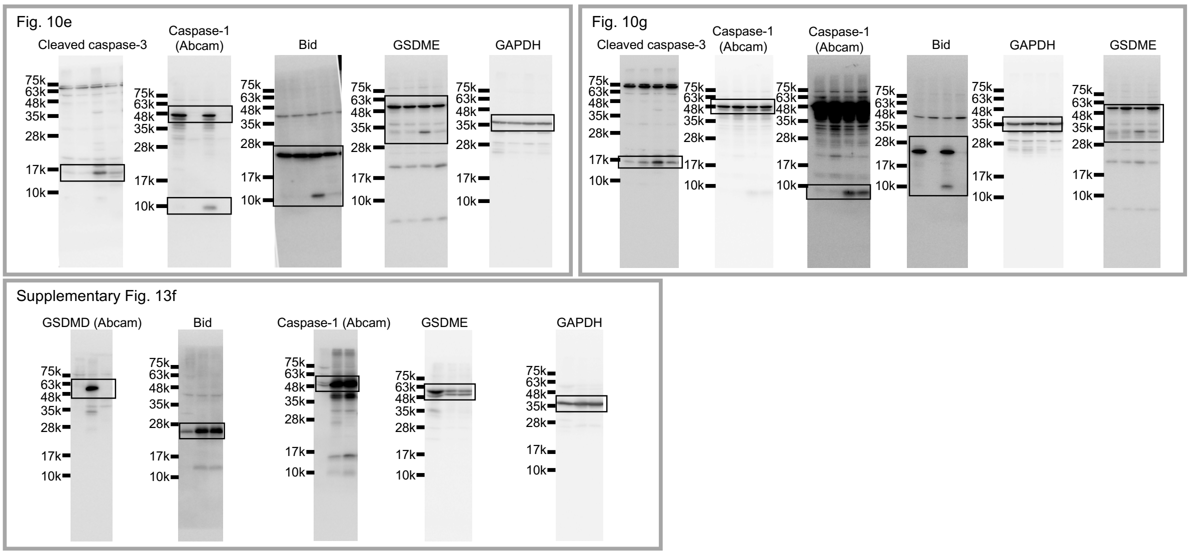

Supplementary Fig. 16 Uncropped scans of western blots.

Supplementary Movie 1 Pyroptotic cell death induced by iCaspase-1. WT CL26-iCasp1 cells were treated with 50 nM AP20187 in the presence of Yo-Pro-1. Yo-Pro-1 fluorescence is shown in green. Scale bar, 50 µm.

Supplementary Movie 2 Apoptosis and secondary necrosis induced by iCaspase-1. *Gsdmd*-KO CL26-iCasp1 cells were treated with 50 nM AP20187 in the presence of Yo-Pro-1. Yo-Pro-1 fluorescence is shown in green. Scale bar, 50 µm.

Supplementary Movie 3 Apoptosis induced by iCaspase-1. *Gsdmd*/*Gsdme*-DKO CL26-iCasp1 cells were treated with 50 nM AP20187 in the presence of Yo-Pro-1. Yo-Pro-1 fluorescence is shown in green. Scale bar, 50 µm.
