# Supplementary material for: Caspase-1 initiates apoptosis in the absence of gasdermin D
Source: Nat Commun. 2019 May 7;10:2091. doi: 10.1038/s41467-019-09753-2 (PMC6505044; doi:10.1038/s41467-019-09753-2)
Supplement: Supplementary file 6 — Reporting Summary [file 41467_2019_9753_MOESM6_ESM.pdf]

nature research

Corresponding author(s): Takashi Suda and Kohsuke Tsuchiya

Last updated by author(s): Mar 20, 2019

Reporting Summary

Nature Research wishes to improve the reproducibility of the work that we publish. This form provides structure for consistency and transparency in reporting. For further information on Nature Research policies, see Authors & Referees and the Editorial Policy Checklist.

Statistics

For all statistical analyses, confirm that the following items are present in the figure legend, table legend, main text, or Methods section.

n/a	Confirmed	
☐	☒	The exact sample size (*n*) for each experimental group/condition, given as a discrete number and unit of measurement
☐	☒	A statement on whether measurements were taken from distinct samples or whether the same sample was measured repeatedly
☐	☒	The statistical test(s) used AND whether they are one- or two-sided<br>*Only common tests should be described solely by name; describe more complex techniques in the Methods section.*
☒	☐	A description of all covariates tested
☐	☒	A description of any assumptions or corrections, such as tests of normality and adjustment for multiple comparisons
☐	☒	A full description of the statistical parameters including central tendency (e.g. means) or other basic estimates (e.g. regression coefficient) AND variation (e.g. standard deviation) or associated estimates of uncertainty (e.g. confidence intervals)
☐	☒	For null hypothesis testing, the test statistic (e.g. *F*, *t*, *r*) with confidence intervals, effect sizes, degrees of freedom and *P* value noted<br>*Give P values as exact values whenever suitable.*
☒	☐	For Bayesian analysis, information on the choice of priors and Markov chain Monte Carlo settings
☒	☐	For hierarchical and complex designs, identification of the appropriate level for tests and full reporting of outcomes
☒	☐	Estimates of effect sizes (e.g. Cohen's *d*, Pearson's *r*), indicating how they were calculated

Our web collection on statistics for biologists contains articles on many of the points above.

Software and code

Policy information about availability of computer code

Data collection	FACSDiva
Data analysis	FlowJo V10, GraphPad Prism 6.0

For manuscripts utilizing custom algorithms or software that are central to the research but not yet described in published literature, software must be made available to editors/reviewers. We strongly encourage code deposition in a community repository (e.g. GitHub). See the Nature Research guidelines for submitting code & software for further information.

Data

Policy information about availability of data

All manuscripts must include a data availability statement. This statement should provide the following information, where applicable:

- Accession codes, unique identifiers, or web links for publicly available datasets
- A list of figures that have associated raw data
- A description of any restrictions on data availability

All data supporting the findings of this study are available within the article and its Supplementary Information files or are available from the corresponding authors upon reasonable request. The source data for creating columns and plots in Figs 1-10 and Supplementary Figs 2-13, 15 are provided as a Source Data file. Uncropped scans of western blots are shown in Supplementary Fig. 16.

Field-specific reporting

Please select the one below that is the best fit for your research. If you are not sure, read the appropriate sections before making your selection.

☒ Life sciences ☐ Behavioural & social sciences ☐ Ecological, evolutionary & environmental sciences

For a reference copy of the document with all sections, see nature.com/documents/nr-reporting-summary-flat.pdf

Life sciences study design

All studies must disclose on these points even when the disclosure is negative.

Sample size	Sample size was determined according to our experience and literature reporting similar experiments.
Data exclusions	No sampe was excluded.
Replication	All attempts at replication were successful.
Randomization	Microscopic images of the cells were randomly selected from sample cultures.
Blinding	Most of Western blotting analyses were performed by an experimenter who was blinded to the samples.

Reporting for specific materials, systems and methods

We require information from authors about some types of materials, experimental systems and methods used in many studies. Here, indicate whether each material, system or method listed is relevant to your study. If you are not sure if a list item applies to your research, read the appropriate section before selecting a response.

Materials & experimental systems

n/a	Involved in the study	
☐	☒	Antibodies
☐	☒	Eukaryotic cell lines
☒	☐	Palaeontology
☐	☒	Animals and other organisms
☒	☐	Human research participants
☒	☐	Clinical data

Methods

n/a	Involved in the study	
☒	☐	ChIP-seq
☐	☒	Flow cytometry
☒	☐	MRI-based neuroimaging

Antibodies

Antibodies used	Cleaved Caspase3 (#9661, 1:1000), Caspase-3 (#9665, 1:1000), Caspase-6 (#9762, 1:500), Caspase-7 (#12827, 1:500), cleaved Caspase-8 (#8592, 1:500), Caspase-8 (#4790, 1:1000), Caspase-9 (#9504, 1:1000), Bid (#2003, 1:500), RIPK3 (#15828, 1:1000), and Cytochrome c (#4272, 1:500), Apaf-1 (#8969, 1:1000) from Cell Signaling Technology; MTCO1 (ab14705, 1:1000), GSDME (ab215191, 1:1000), GSDMD (ab209845, 1:1000), Caspase-1 (ab179515, 1:1000) from Abcam; Caspase-1 (sc-514, 1:1000) and GSDMD (sc-393581, 1:400) from Santa Cruz Biotechnology; Caspase-2 (MAB3507, 1:500) from Merck; and GAPDH (M171-3, 1:1000) from Medical & Biological Laboratories; TruStain fcX (101320), APC anti-mouse CD117 (105811), FITC anti-mouse FcεRIα (134305) from BioLegend.
Validation	Specificity of most of antibodies was validated using wild-type and knockout cells. All commercial antibody validations are available on manufacturers' websites.

Eukaryotic cell lines

Policy information about cell lines

Cell line source(s)	Colon-26 cells (RIKEN BioResource Center), RAW264.7 cells (Institute of Medical Science, University of Tokyo), L929 cells (Cell Resource Center for Biomedical Research, Institute of Development, Aging and Cancer, Tohoku University)
Authentication	No authentication procedures were used.
Mycoplasma contamination	All the cell lines are negative for mycoplasma contamination.
Commonly misidentified lines (See ICLAC register)	These cell lines are not on the list of commonly misidentified lines.

Animals and other organisms

Policy information about studies involving animals; ARRIVE guidelines recommended for reporting animal research

Laboratory animals	Wild-type C57BL/6 mice were purchased from Japan SLC. Gsdmd-/- mice, Casp1-/- mice, Pycard-/- mice, and Bid-/- mice had been backcrossed to C57BL/6 mice. Gsdmd-/- Bid-/- mice were generated by crossing Gsdmd-/- mice and Bid-/- mice. Mice were bred and maintained in pathogen-free animal facilities at Kanazawa University, and were used between 10 – 20 weeks of age. Bone marrow preparations were from age- and sex-matched mice.
Wild animals	N/A
Field-collected samples	N/A
Ethics oversight	All animal experiments were approved by the Animal Care and Use Committee of Kanazawa University (AP-143305, AP-173853, AP-184013), and conducted in accordance with the Kanazawa University Animal Experimentation Regulations and the International Guiding Principles for Biomedical Research Involving Animals by the Council for International Organization of Medical Sciences and the international council for Laboratory animal science (December 2012).

Note that full information on the approval of the study protocol must also be provided in the manuscript.

Flow Cytometry

Plots

Confirm that:

☒ The axis labels state the marker and fluorochrome used (e.g. CD4-FITC).

☒ The axis scales are clearly visible. Include numbers along axes only for bottom left plot of group (a 'group' is an analysis of identical markers).

☒ All plots are contour plots with outliers or pseudocolor plots.

☒ A numerical value for number of cells or percentage (with statistics) is provided.

Methodology

Sample preparation	CL26-iCasp1 cells were collected by trypsinization and pelleted by centrifugation at 800 x g for 3 min. The cells were stained with annexin V-Cy5 (BioVision, 1013; 1:1000 dilution) and propidium iodide (Immuno Chemistry Technologies, 638; 1:500 dilution) in annexin V binding buffer containing 10 mM HEPES (pH 7.4), 150 mM NaCl, 5 mM KCl, 1 mM MgCl2, and 1.8 mM CaCl2 for 15 min on ice. To assess mitochondrial membrane potential, cells were stained with 100 nM tetramethylrhodamine methlester (TMRM; Thermo Fisher Scientific, T668) in culture medium for 15 min room temperature. Bone marrow-derived mast cells suspended in staining buffer (PBS, 1% BSA, 0.1% NaN3) were pretreated with TruStain fcX (BioLegend, 101320) at 4 °C for 30 min and then stained with APC anti-mouse CD117 (BioLegend, 105811) and FITC anti-mouse FcεRIα (BioLegend, 134305) at 4 °C for 30 min.
Instrument	FACSCanto II flow cytometer (BD Biosciences)
Software	FACSDiva and FlowJo
Cell population abundance	N/A
Gating strategy	Gating was performed once using light scatter properties to exclude cellular debris and clumped cells as shown in Fig. S3c.

☒ Tick this box to confirm that a figure exemplifying the gating strategy is provided in the Supplementary Information.
